# Phylogeographic analysis of mitochondrial genome polymorphism in the Shors

**DOI:** 10.18699/vjgb-26-75

**Published:** 2026-09

**Authors:** B.A. Malyarchuk, G.A. Denisova, A.N. Litvinov, N.V. Pokhilyuk

**Affiliations:** Institute of Biological Problems of the North of the Far Eastern Branch of the Russian Academy of Sciences, Magadan, Russia; Institute of Biological Problems of the North of the Far Eastern Branch of the Russian Academy of Sciences, Magadan, Russia; Institute of Biological Problems of the North of the Far Eastern Branch of the Russian Academy of Sciences, Magadan, Russia; Institute of Biological Problems of the North of the Far Eastern Branch of the Russian Academy of Sciences, Magadan, Russia

**Keywords:** mitochondrial genome, phylogeographic analysis, human populations, Siberia, Shors, митохондриальный геном, филогеографический анализ, популяции человека, Сибирь, шорцы

## Abstract

The variability of nucleotide sequences of complete mitochondrial genomes in the Shors, a Turkic-speaking people from the Altai–Sayan region, was studied. The Shors exhibit a high level of nucleotide diversity due to the presence of two components of the East and West Eurasian origin in their gene pool (79.5 and 20.5 %, respectively). However, a relatively low level of mtDNA haplotype diversity was found among the Shors, which is associated with the high prevalence of certain identical mitochondrial haplotypes. Multidimensional scaling of interpopulation genetic distances indicates that the Shors fall in between East Asian ethnic groups and indigenous East Siberian peoples. By examining the polymorphism of whole mitochondrial genomes in individuals with the same hypervariable segment 1 haplotype belonging to haplogroup F1b1, found in ~37 % of the Shors, we identified six mtDNA haplotypes belonging to haplogroups F1b1-b1 and F1b1-b2. Phylogeographic analysis shows that the Shor mitochondrial gene pool is characterized by a high frequency (65.9 %) of mtDNA haplogroups found solely in the Altai–Sayan region: A-a1c1a, B4b1a3a1a, C4a1a4a1a, C4e, C5c*, D4b1a2a1a, D4m2a2, D5c1a2b, F1b1-b1, F1b1-b2, F2b1b1, and H8b1a. According to molecular dating results, the evolutionary age of these mtDNA haplogroups varies from 0.9 to 5.0 thousand years. All of the mtDNA haplogroups listed are of East Asian origin, except for the West Eurasian H8b1a. The isolated evolution of mitochondrial haplogroups specific to the peoples of the Altai–Sayan region is assumed to have been facilitated by their isolation from East Asian neighbors. This isolation occurred during the Bronze Age, when the Afanasyevo culture formed as a result of the mixing of indigenous populations and migrants – representatives of the Yamnaya culture, which existed in the Urals and Volga regions. In the absence of a significant influx of additional East Asian mtDNA haplotypes to the populations of the Altai–Sayan region over several thousand years, it appears that existing genetic lineages of East Asian origin diversified.

## Abstract (ru)

Получены данные об изменчивости нуклеотидных последовательностей целых митохондриальных геномов у шорцев – тюркоязычного народа Алтае-Саянского региона. У шорцев выявлен высокий уровень нуклеотидного разнообразия, обусловленный наличием в генофонде двух компонентов восточно- и западноевразийского происхождения (79.5 и 20.5 % соответственно). При этом отмечен относительно низкий (в масштабах Сибири) уровень разнообразия гаплотипов мтДНК, что связано с высокой распространенностью у шорцев некоторых одинаковых митохондриальных гаплотипов. Результаты многомерного шкалирования межпопуляционных генетических дистанций свидетельствуют о том, что шорцы занимают промежуточное положение между этническими группами Восточной Азии и коренными народами Восточной Сибири. Исследование полиморфизма целых митохондриальных геномов у индивидуумов, характеризующихся одинаковым гаплотипом гипервариабельного сегмента 1, который относится к гаплогруппе F1b1 и обнаружен у ~37 % шорцев, позволило определить 6 гаплотипов мтДНК, принадлежащих к гаплогруппам F1b1-b1 и F1b1-b2. Результаты филогеографического анализа показали, что особенностью митохондриального генофонда шорцев является высокая частота (65.9 %) гаплогрупп мтДНК, которые распространены исключительно в популяциях Алтае-Саянского региона: A-a1c1a, B4b1a3a1a, C4a1a4a1a, C4e, C5c*, D4b1a2a1a, D4m2a2, D5c1a2b, F1b1-b1, F1b1-b2, F2b1b1, H8b1a. Эволюционный возраст этих гаплогрупп мтДНК согласно результатам молекулярного датирования варьирует от 0.9 до 5.0 тыс. лет. Все указанные выше гаплогруппы мтДНК восточноазиатские по происхождению, за исключением западноевразийской H8b1a. Обособленному развитию митохондриальных гаплогрупп, специфичных для народов Алтае-Саянского региона, возможно, способствовала изоляция от восточноазиатских соседей, вызванная формированием афанасьевского населения в эпоху бронзового века в результате смешения популяций коренных жителей региона и мигрантов – представителей ямной культурно- исторической общности, существовавшей в Приуралье и Поволжье. В условиях отсутствия интенсивного притока дополнительных восточноазиатских гаплотипов мтДНК в течение нескольких тысяч лет в популяциях Алтае-Саянского региона, по всей видимости, произошла диверсификация уже имевшихся генетических линий восточноазиатского происхождения.

## Introduction

Studies of mitochondrial DNA (mtDNA) polymorphisms have demonstrated that the gene pools of most ethnic groups in Western Siberia contain significant contributions from West Eurasian mtDNA lineages. This component is most prevalent (comprising 20–40 % of the gene pool) among modern populations in the Altai–Sayan region, including the Khakass, Shors, Teleuts, Altai-Kizhi, Telengits and Altai Kazakhs (Derenko et al., 2003; Derenko, Malyarchuk, 2010; Gubina et al., 2013). Paleogenomic studies have shown that the Neolithic population of Altai and neighboring regions primarily consisted of East Asian mtDNA lineages, although some West Eurasian mtDNA lineages, such as U2e1, were already present in the region approximately 7.5 thousand years ago (Wang K. et al., 2023; Gill et al., 2025). The influx of Eastern European populations into southwestern Siberia and Mongolian steppes during the Bronze Age (5.0–4.0 thousand years ago) led to a significant increase in the proportion of European genetic components in the gene pool of the ancient population (Allentoft et al., 2015). However, about 2.0 thousand years ago, the expansion of the Xiongnu caused a significant increase in the proportion of East Asian mtDNA haplotypes in the gene pools of the peoples of Central Asia and the Altai–Sayan region (Damgaard et al., 2018; Jeong et al., 2020).

The Shors are an indigenous people inhabiting Mountain Shoria in southern Kemerovo Oblast. They speak a language belonging to the Uyghur group of Turkic languages. According to ethnographic data, they are related to other ethnic groups of the Northern Altai: the Chelkans, Kumandins, and Tubalars. In anthropological terms, however, the Shors and Kumandins exhibit similarities to certain Ugric and Samoyedic peoples, such as the Khanty, Mansi, and Selkups, who are characterized by the Uralic anthropological type (Moiseev, 1999). The Shors appear to be Turkicized descendants of the Ugric-, Samoyedic-, and Ket-speaking populations of the northern taiga region of the Altai–Sayan Highlands (Peoples of Siberia, 1956). According to the 2021 Russian Federation census, there are 10,500 Shors.

An analysis of hypervariable segment 1 (HVS1) of the mitochondrial genome has shown that the Shors inhabiting the Altai and Sayan Mountains are genetically similar to other ethnic groups in the region, including the Khakass, Teleuts, Altai- Kizhi, Tuvinians, Todjins, and Tofalars (Derenko, Malyarchuk, 2010; Gubina et al., 2022). However, a phylogenetic analysis of HVS1 nucleotide sequences shows that the Shors are somewhat distant from the main cluster of Altai–Sayan populations (Derenko, Malyarchuk, 2010). This distance may be due to their distinct anthropological composition and their presumed kinship with Ugric and Samoyedic peoples. This hypothesis is supported by the presence of certain mitochondrial lineages (J1b1 and U4b1) in the mitochondrial gene pool of the Shors, which are characteristic of indigenous populations in West Siberia and the Volga-Ural region (Derenko, Malyarchuk, 2010).

An interesting feature of the mitochondrial gene pool of the Shors, as well as the related Khakass people, is the high prevalence of mtDNA haplotypes belonging to the East Asian haplogroup F (ranging from 15 to 40 %), although their frequencies among other Siberian ethnic groups are significantly lower (Derbeneva et al., 2002; Derenko et al., 2007; Gubina et al., 2013). The greatest diversity of mitochondrial lineages within haplogroup F is found in Southeast Asian populations (Hill et al., 2007).

The gene pools of the Shor and Khakas peoples are dominated by F1b mtDNA lineages, which originated in southern China (Hill et al., 2007). One of these F1b haplotypes, with the 16189-16232CA-16249-16304-16311 HVS1 motif, is widespread among the Shors (up to 37 %) and the Khakass (up to 26 %) (Derenko et al., 2007; Gubina et al., 2013). It is believed that this is due to the “founder effect” that occurred when these populations formed (Derenko, Malyarchuk, 2010). Due to the low resolution of mtDNA HVS1 polymorphism analysis, it is reasonable to employ a more informative method, such as analysis of nucleotide sequence variability in whole mitochondrial genomes. This will clarify the extent to which previously identified F1b mtDNA HVS1 sequences in the Shors are similar. Additionally, analyzing the polymorphism of the entire mitochondrial genome of the Shors at the population level would provide the most comprehensive understanding of their gene pool diversity and enable us to trace the phylogenetic relationships among mtDNA haplotypes using phylogeographic analysis. This work aims to study these issues.

## Materials and methods

Whole blood samples were taken from 33 Shors chosen from residents of the Tashtagol District of the Kemerovo Region. The Regional Ethics Committee for Medical and Biological Research under the Presidium of the Far Eastern Scientific Center of the Russian Academy of Sciences, Magadan, approved the study (Protocol No. 001/011, January 21, 2011).

We determined the nucleotide sequences of whole mitochondrial genomes by Sanger DNA sequencing on an ABI 3500xL genetic analyzer (Applied Biosystems), as in our previous work (Derenko, Malyarchuk, 2010). Amplification products were sequenced using the BrilliantDye™ Terminator Cycle Sequencing kit v3.1 (Netherlands). The SeqScape 2.7 software package (Applied Biosystems) was used for alignment and assembly of the mitochondrial genomes. The nucleotide sequences of the Shors’ mitochondrial genomes have been deposited in GenBank (www.ncbi.nlm.nih.gov) under accession numbers PX641521–PX641539. The analysis also utilized 11 previously sequenced mitochondrial genomes from Shors in the Tashtagol District of the Kemerovo Region (Table S1)1.

Supplementary Materials are available in the online version of the paper: https://vavilov.elpub.ru/jour/manager/files/Suppl_Mal_Engl_30_5.xlsx


We performed phylogenetic analysis of mitochondrial genome nucleotide sequences and estimated the evolutionary age of mtDNA monophyletic clusters using the mtPhyl v4.015 software package (https://isogg.org/wiki/MtPhyl), as previously described (Malyarchuk et al., 2025). We used the phylogenetic classifications proposed by the PhyloTree (www.phylotree.org) and YFull MTree (https://www.yfull.com/mtree/) resources to determine mtDNA haplogroups.

Our phylogeographic analysis of mtDNA involved data on the variability of complete mitochondrial genomes from various human populations as reported in GenBank, the Logan DNA Project (http://www.ianlogan.co.uk), and YFull MTree. As of November 2025, GenBank contains over 62,500 mitochondrial genomes from individuals of different ethnic backgrounds worldwide (https://www.mitomap.org/foswiki/ bin/view/MITOMAP/GBFreqInfo). The ethnic affiliation of the studied samples was determined based on information provided in the databases.

A comparative analysis of mtDNA variation at the interethnic level is based on 2,923 mitochondrial genomes from various East Asian populations. These include Han Chinese and Japanese (Zheng et al., 2011), Evenks, Yakuts, and Evens from Yakutia, Nivkhs, Udegeys, and Yukaghirs (Duggan et al., 2013), Koryaks and Evens from the Magadan region, Chukchi (Derenko et al., 2023), Buryats (Derenko et al., 2018), Mongols, Barghuts, and Khamnigans (Derenko et al., 2022), Uyghurs (Zheng et al., 2017), Tajiks and Kyrgyz (Peng et al., 2018), Daurs (Wang C.-Z. et al., 2022). We used the DnaSP 5.10.01 software package (Librado, Rozas, 2009) to calculate genetic diversity parameters in the populations. We performed an analysis of molecular variance (AMOVA, Fst analysis) based on pairwise nucleotide differences between mitogenomes using the Arlequin 3.5.1.2 software package (Excoffier, Lischer, 2010). We used the multidimensional scaling method of interpopulation Fst differences (STATISTICA 10 software package, StatSoft Inc.) to investigate the distri1 bution of populations in two-dimensional space. We calculated interpopulation genetic distances (DA и DXY) using the equation

**Formula. 1.**
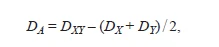
Formula. 1

where DXY is the average number of nucleotide differences between populations X and Y; DX and DY are the average numbers of nucleotide differences within populations X and Y, respectively (Nei, 1987).

## Results and discussion

**Diversity of the mitochondrial gene pool among the Shor people and genetic differentiation of East Asian populations**

Analysis of mitochondrial genome nucleotide sequence polymorphisms encompassing the Shors (33 new samples and 11 previously published ones) and other ethnic groups in Siberia, Central Asia, and East Asia showed that the mitochondrial haplotypes belong to 23 haplogroups (Table 1 and Table S1).

**Table 1.**
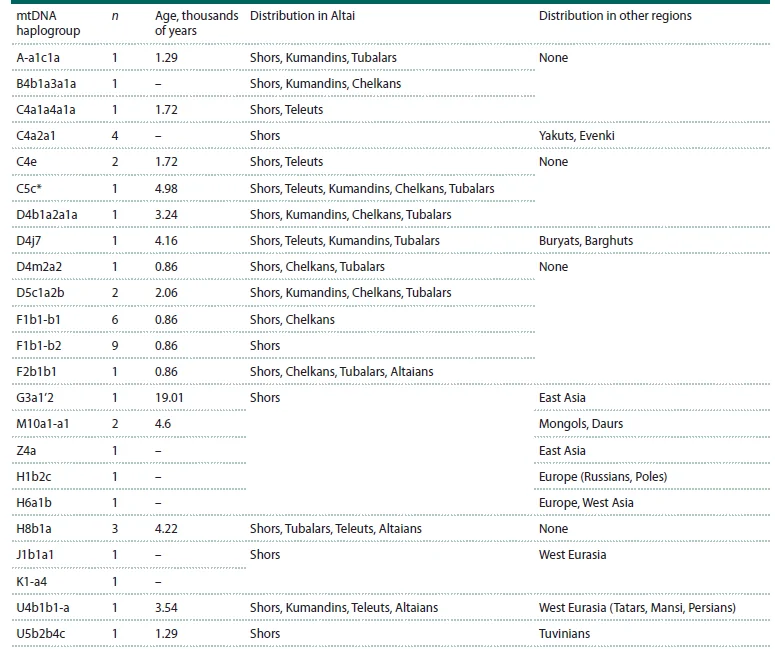
mtDNA haplogroups of the Shors, their evolutionary age, and geographic distribution Notе. n – the number of samples belonging to the haplogroup. Dashes (–) indicate cases where the evolutionary age cannot be determined because of data shortage.

Compared to other East Asian populations, the Shor sample studied is characterized by relatively low haplotype diversity (H = 0.979), comparable to data from populations in Northeast Siberia, such as the Chukchi, Koryaks, Yukaghirs, and Nivkhs (Derenko et al., 2023). This is apparently due to the high prevalence of identical haplotypes among the Shors, which mainly belong to haplogroup F1b1 (Table 1). At the same time, the nucleotide diversity among the Shors is at its maximum level, with π = 0.00236 and an average number of pairwise nucleotide differences of k = 39.01. Previous studies have shown that similar nucleotide diversity values are found among the Daurs, Kyrgyz, and Khamnigans (Derenko et al., 2023).

An analysis of genetic differences based on the number of nucleotide substitutions per site between populations (DXY and DA) showed the least genetic differences of Shors from the Han, Uyghurs, Mongols, and Kyrgyz (Table 2). The results of Fst-analysis also demonstrated minimal differences between the Shors and the Mongols, Han, Kyrgyz, and Uyghurs (Fst values of 5.5–6.0 %), while Fst differences between the Shors and Paleo-Asian populations (Chukchi, Koryaks, and Nivkhs) exceeded 20 % (Table S2).

**Table 2.**
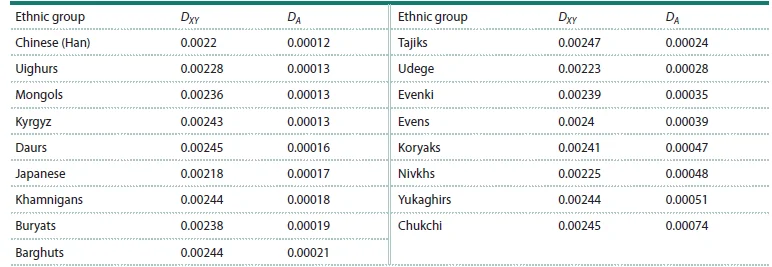
Interpopulation genetic distances (DXY and DA are numbers of substitutions per site between populations) between the Shors and other East Asian ethnic groups Notе. The distance values are ranked by DA.

Our analysis of interpopulation Fst differences conducted using multidimensional scaling showed that the Shors are situated between the compactly clustered East Asian ethnic groups and the scattered Eastern Siberian populations, as illustrated in Figure 1 in a two-dimensional genetic space. In the latter group, only the Evens, Evenki, Koryaks, and Yukaghirs show a tendency toward clustering. Unfortunately, the Samoyedic and Ugric groups are absent from the populations studied. This is due to the lack of population-level data on whole mitochondrial genome variability for many ethnic groups in Siberia and Eastern Europe. Therefore, a more detailed study of the origins of the Shors – for instance, based on the mixing of Samoyedic and Ugric tribes with Turkic-speaking groups – remains to be conducted after databases on mtDNA variation among indigenous Siberian and neighboring populations are expanded.

**Fig. 1.**
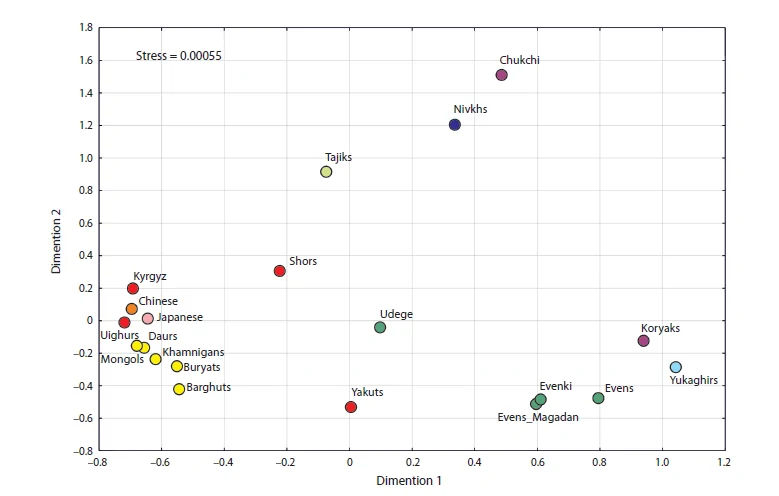
Results of multidimensional scaling of interpopulation Fst values based on pairwise nucleotide differences between whole mitochondrial genomes in populations from Siberia, East Asia, and Central Asia. The colors indicate the linguistic affiliation of the populations: red for the Turkic group of the Altaic family; yellow for the Mongolic group of the Altaic family; green for the Tungusic group of the Altaic family; purple for the Chukotko-Kamchatkan family; orange for the Sino-Tibetan family; pale pink for the Japanese language; light yellow for the Indo-Iranian group of the Indo-European family (Tajiks), and other colors for linguistic isolates (Nivkhs and Yukaghirs).

**Diversity of F1b1 haplotypes in the Shors**

Previous studies have shown that 30 of the 82 Shor individuals examined (36.6 % of the sample) exhibit the HVS1 haplotype 16189-16232CA-16249-16304-16311, belonging to haplogroup F1b1 (Derenko et al., 2007). In this study, we sequenced the entire mitochondrial genomes of 15 Shor individuals carrying this haplotype. Our study demonstrated that the HVS1 haplotype belongs to two haplogroups: F1b1-b1 and F1b1-b2. The former has already been recorded among the Chelkans and Khakass, according to the YFull MTree database. The latter is a new haplogroup, which we have identified only among the Shors (Fig. 2). The evolutionary age of both haplogroups is 0.86 thousand years. However, if the age is estimated without considering identical samples, i. e., based solely on haplotypes, it increases to 1.72 (0–4.13) thousand years for both.

**Fig. 2.**
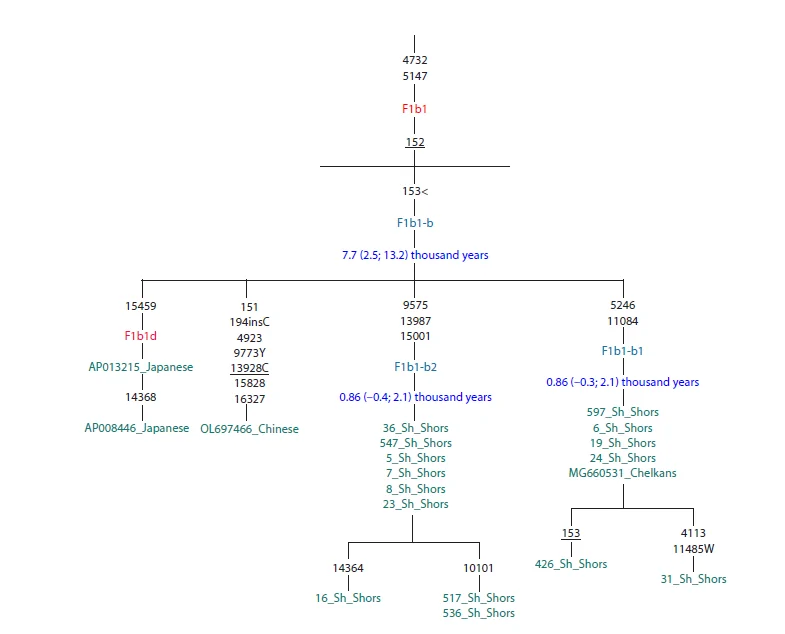
Phylogenetic tree of mtDNA haplogroup F1b1-b. The branches indicate the nucleotide positions where transitions occurred. For transversions and cases of heteroplasmy, the nucleotides are labeled. “Ins” denotes a nucleotide insertion. Nucleotide positions where reverse mutations were recorded are underlined, and recurrent mutations are marked with the symbol “<”. GenBank accession numbers and ethnic affiliation are shown.

At the same time, haplogroup F1b1-b is approximately 7.7 thousand years old, and it includes mitochondrial lineages found among Japanese and Chinese peoples (Fig. 2). This suggests that F1b1-b mtDNA lineages were introduced into the Altai peoples’ gene pools from East Asia quite some time ago. Conversely, the high prevalence of a few F1b1-b mtDNA haplotypes in the Shor population could be due to a founder effect in their demographic history.

**The high population specificity of Altai mtDNA variants**

The results of the phylogeographic analysis show that the mitochondrial gene pool of the Shor sample is predominantly composed of East Asian haplotypes (79.5 %) (Table 1). The West Eurasian mitochondrial lineages identified in the Shors are represented by single haplotypes (H1b2c, H6a1b, J1b1a1, and K1-a4) or subclades (U4b1b1-a and U5b2b4c) of relatively recent evolutionary origin (within the last 3.5 thousand years). However, some subclades of West Eurasian mtDNA haplotypes evolved over a relatively long period within the gene pools of Siberian peoples (Table 1, Table S1). One example is haplogroup U4b1b1-a, which is primarily found in the Altai region but has also been observed in populations in the Volga region and Iran. All other U4b1b1 subgroups have been identified in European populations. Haplogroup U5b2b4 is primarily found in European populations, but its subgroup, U5b2b4c, has been identified among the Shors and Tuvinians. Haplogroup H8b is of particular interest because its distribution is associated with Western Eurasia (subgroups H8b2 and H8b3). However, the most diverse subgroup is H8b1, which has been recorded in various Siberian and Central Asian populations. Its evolutionary age is estimated to be between 8.0 and 9.5 thousand years old (Fig. S1) (Derenko et al., 2014), which points to a long-standing presence of the H8b1 haplogroup in eastern North Eurasia. The H8b1a subgroup is primarily found in the Altai region.

A distinctive feature of the mitochondrial gene pool of the Shors is the high frequency (65.9 %) of mtDNA haplogroups that are found exclusively in the Altai region: A-a1c1a, B4b1a3a1a, C4a1a4a1a, C4e, C5c*, D4b1a2a1a, D4m2a2, D5c1a2b, F1b1-b1, F1b1-b2, F2b1b1, and H8b1a. According to molecular dating results, their evolutionary ages range from 0.86 to 4.98 thousand years (Table 1, Fig. S1). All of the aforementioned mtDNA haplogroups are of East Asian origin, except for H8b1a.

## Conclusion

Archaeological, anthropological, and paleogenomic data show that the emergence of the Afanasyevo archaeological culture in the Altai and the steppes of the Minusinsk Basin during the Bronze Age (approximately about 5.0 thousand years ago) was associated with the migration of the Yamnaya culture population from the Volga-Ural region (Allentoft et al., 2015; Jeong et al., 2020). This population was characterized by the Proto-European anthropological type and genetic lineages of West Eurasian origin. This explains the presence of a wide variety of West Eurasian mtDNA haplotypes among the populations of the Altai–Sayan region. It is of particular interest, though, that the component of the Shor mitochondrial gene pool specific to Altai peoples is mainly represented by East Asian mitochondrial lineages. Their evolutionary age, approximately up to 5.0 thousand years ago, falls within the period of isolation from East Asian neighbors’ gene pools caused by the formation of the mixed Afanasyevo population. In the absence of a significant influx of additional East Asian haplotypes over the course of several thousand years, it appears that the existing genetic lineages of East Asian origin began to diversify within the populations of the Altai–Sayan region. The isolated development of mitochondrial haplogroups may have also been facilitated by the local natural features, as the Altai–Sayan mountain range could have served as a refuge for preserving “relict” gene pools and as a regulator of human migration patterns (Balanovska et al., 2014).

The study of the variability in full-length mtDNA nucleotide sequences demonstrates that the mitochondrial gene pool of the Shors is characterized by a high frequency (65.9 %) of a genetic component specific to ethnic groups inhabiting Altai, such as the Kumandins, Chelkans, Tubalars, and Teleuts. This component is primarily represented by mitochondrial lineages of East Asian origin. These lineages diversified during the formation of a mixed population based on the indigenous Altai populations and migrants from the Yamnaya cultural-historical community, which existed in the Ural and Volga regions.

## Conflict of interest

The authors declare no conflict of interest.
